# Three-dimensional direct cell patterning in collagen hydrogels with near-infrared femtosecond laser

**DOI:** 10.1038/srep17203

**Published:** 2015-11-25

**Authors:** Kolin C. Hribar, Kyle Meggs, Justin Liu, Wei Zhu, Xin Qu, Shaochen Chen

**Affiliations:** 1 Department of NanoEngineering, University of California San Diego, La Jolla, CA, 92093-0448; 2 Materials Science and Engineering Program, University of California San Diego, La Jolla, CA.

## Abstract

We report a methodology for three-dimensional (3D) cell patterning in a hydrogel *in situ*. Gold nanorods within a cell-encapsulating collagen hydrogel absorb a focused near-infrared femtosecond laser beam, locally denaturing the collagen and forming channels, into which cells migrate, proliferate, and align in 3D. Importantly, pattern resolution is tunable based on writing speed and laser power, and high cell viability (>90%) is achieved using higher writing speeds and lower laser intensities. Overall, this patterning technique presents a flexible direct-write method that is applicable in tissue engineering systems where 3D alignment is critical (such as vascular, neural, cardiac, and muscle tissue).

Tissue engineering offers the ability to generate functional tissues for implantation and *in vitro* modeling^1^. Three-dimensional (3D) cellular organization is critical to the function of a given tissue, and moreover, 3D cell alignment in tissues such as vasculature, neural, cardiac, and muscle, plays an integral role in their overall function^2^,^3^,^4^,^5^,^6^. Thus, methods that enable 3D cellular patterning and direct 3D tissue organization are of high importance^7^,^8^. Hydrogels – water-swollen polymer networks - are extensively used as the scaffolding in engineered tissues^9^. There are various ways to control hydrogel structure in 3D for the purpose of dictating cellular organization and alignment. 3D printing, which includes extrusion, stereolithography, and projection printing, builds hydrogel structures in an additive fashion^10^,^11^,^12^. On the other hand, the selective removal of material from a bulk gel (for instance, using a ultrafast laser) can also generate 3D structures with precise architecture^13^,^14^.

To this end, we have developed a novel patterning method that controls the spatial organization of cells in a 3D collagen hydrogel. The hydrogel, composed of gold nanorods and encapsulated cells, reacts to a focused near-infrared (NIR) laser beam to thermally denature the collagen in a computer-directed pattern. Nanorods absorb NIR light and convert this energy to heat – called the photothermal effect – thus, stimulating thermal denaturation of the surrounding collagen matrix (Fig. 1). Photothermal activation of temperature-sensitive materials has previously been explored for triggering drug release, bulk hydrogel degradation, and 2D hydrogel patterning^10^,^15^,^16^. Here, we report for the first time its utility in 3D hydrogel patterning. Patterning takes place within *milliseconds* and can be tuned by the laser writing speed and laser power. Channels produced within the hydrogel cause cell migration and 3D cell alignment to the patterns over a period of 14 days. We hypothesize this is due, in part, to the denaturation and uncrosslinking of the collagen fibrils, which denature at 55 °C. This single-step process has broad applicability in tissue engineering systems requiring 3D cell alignment – including vascular, neural, cardiac, and muscle tissue.

## Results

### Gold Nanorod Characterization

Using transmission electron microscopy (TEM), average length, width, and aspect ratio for the PEG-nanorods was determined to be 41.4 ± 3.5 nm, 10.7 ± 0.6 nm, and 3.86 ± 0.4, respectively (Supplementary Figure S1). These values are in line with previous literature for nanorods that absorb the laser wavelength of 800 nm^17^. Nanorods with this size distribution consisted of >90% of the total gold nanoparticles in solution, with a small fraction existing as gold nanospheres or cubes. Thus, we anticipated that the number of gold nanorods within our collagen hydrogels are similar (or at least within the same order of magnitude) to the evaluated concentration of 5.45 e-9 moles nanorods per liter.

Additionally, we did not anticipate any morphology changes to the gold nanorods due to NIR light exposure since our femtosecond laser operates in nJ energy per pulse range – morphology changes often occur at higher energy levels in the μJ or mJ ranges^18^.

### Collagen Hydrogel Characterization

**Figure 2:**
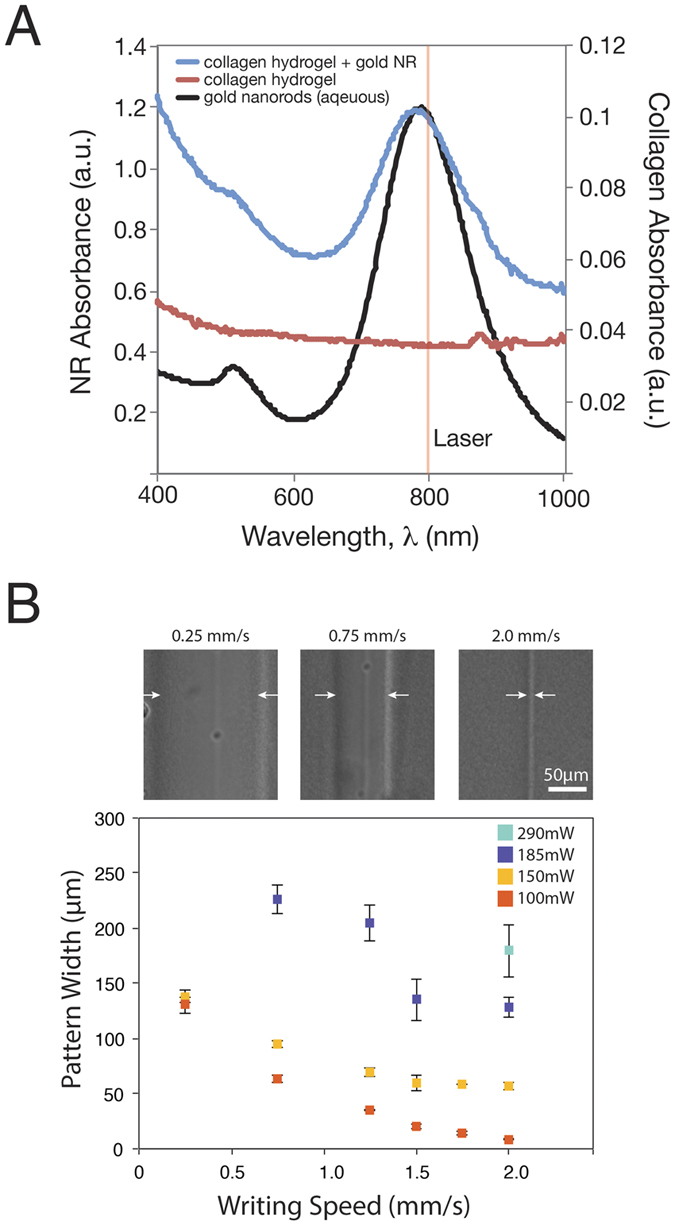
Characterization of hydrogel patterning. (**A**) Absorbance of collagen hydrogels with and without nanorods, compared to nanorods in aqueous solution. The laser wavelength is also highlighted (800 nm). (**B**) Representative brightfield images of the patterned lines at different writing speeds 0.25, 0.75, and 2.0 mm/s at 100 mW laser power. Characterization of patterned line widths in response to various writing speeds (0.25, 0.75, 1.25, 1.5, 1.75, 2.0 mm/s) and laser powers (100, 150, 185, 290 mW)

Collagen hydrogels with gold nanorods displayed an absorbance profile similar to that of the nanorods in solution (Fig. 2A), peaking at 800 nm–coinciding with the wavelength of the NIR femtosecond laser. Collagen hydrogels without nanorods, conversely, showed low absorbance at 800 nm. When exposed to maximum power and the slowest speed (290 mW, 0.25 mm/s), collagen hydrogels without nanorods (collagen – NR) showed no changes in hydrogel morphology and resulting cell organization, suggesting the gold nanorods played a key role in the patterning mechanism (Supplemental Figure S2).

Internal patterning of the collagen-nanorod hydrogel is a modular process, where laser power and writing speed (in millimeters per second, mm/s) can alter the diameter of the resulting channel pattern. Figure 2B displays brightfield images of the resulting patterns in the collagen gel exposed to 100 mW or 150 mW laser power, at laser scanning speeds of 0.25 mm/s, 0.75 mm/s, or 2.0 mm/s. Figure 2B also shows the graphical representation of this data. An inverse relationship exists between the writing speed and pattern width. Laser power determines the threshold pattern size, where 100 mW can generate 8.7 ± 0.5 μm resolution patterns (equal to the diameter of the laser beam) while higher powers such as 150 mW and 190 mW plateaued at 56.7 ± 3.0 μm and 128.6 ± 9.3 μm, respectively. Patterns made with 290 mW power were barely distinguishable as the response in the collagen gel degraded the entire construct, however at a speed of 2.0 mm/s, the resolution was roughly 179.3 ± 23.5 μm. Writing speeds greater than 2.0 mm/s were not possible due to limitations with the automated stage, but presumably the patterns could become even more optically defined with faster speeds.

### Cell Viability Assessment

**Figure 3:**
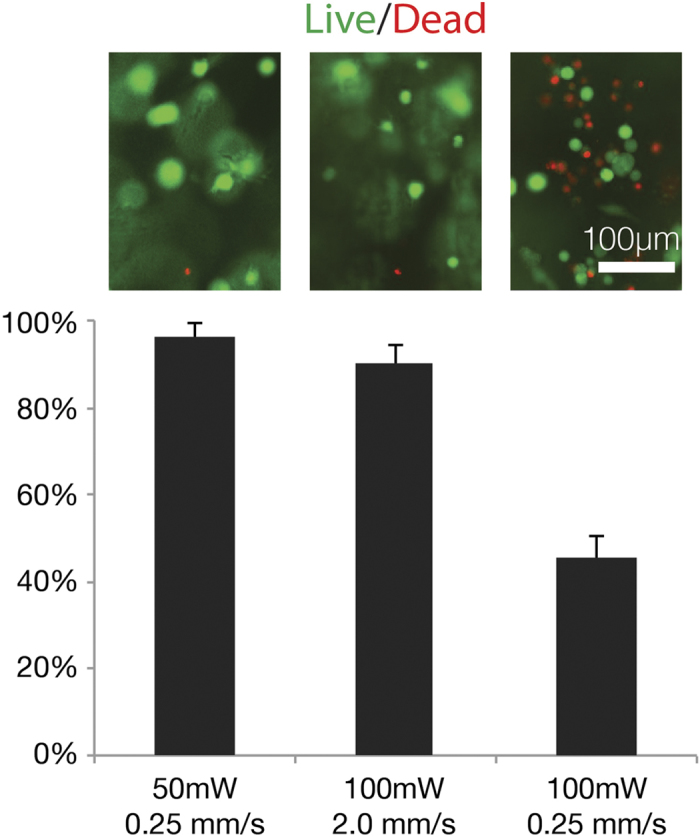
Live (green)/dead (red) fluorescence images and resulting cell viability (live cells as a percent of total cells) according to different writing speeds (mm/s) and laser intensities (mW).

Cell experiments were carried out using bend3 endothelial cells at a concentration of 1000 cells per μl in the collagen-nanorod hydrogels. We patterned channels using various powers and writing speeds and performed a fluorescent live/dead assay with calcein AM/ethidium homodimer to assess cell viability (Fig. 3). Patterns created with higher powers and slower writing speeds produced higher cell death as compared to lower laser power and faster writing speeds. For instance, by keeping the laser power constant at 100 mW and changing the writing speed from 0.25 to 2.0 mm/s (Fig. 3a,b), we were able to increase the cell viability from 45 ± 5% to 90 ± 4%, respectively. By reducing the power to 50 mW at the slower speed of 0.25 mm/s, we increased cell viability to 96 ± 3%, though pattern fidelity was less visualized. Ultimately, we chose a writing speed of 2.0 mm/s and laser power of 100 mW for the remaining cell experiments due to their high pattern fidelity and high cell viability (>90%).

### Patterns Guide 3D Cell Migration, Elongation, and Alignment

**Figure 4:**
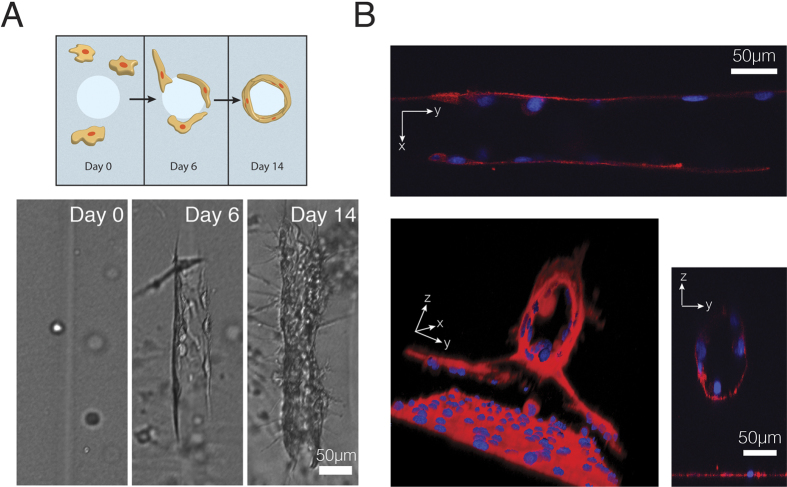
Endothelial cell response to 3D patterning. (**A**) Cell migration and tube formation, visualized in brightfield. (**B**) endothelial tube formation visualized with confocal, showing hollow cores in the YZ plane and aligned endothelial cells in the XY plane.

We allowed collagen gels to incubate for several weeks, during which we observed cell migration towards the patterns, cell elongation on the walls, and by day 14, maturing of this 3D cell alignment (Fig. 4A). We confirmed that the channels were inducing this cellular organization by comparing with hydrogels without patterns (Supplementary Figure S3). It is clearly shown that the patterns induce cellular tube formation.

We hypothesized that the cell elongation was a hollow tube structure, as we were creating channels. Thus after day 14, the gels were fixed and stained for actin and nuclei and imaged using confocal microscopy. Using Volocity 3D reconstruction software (PerkinElmer Inc), we were able to visually observe the tube structure (Fig. 4B), and XY and YZ planes confirmed the tubes length and hollow nature (Fig. 4B outset). These data suggest tube formation occurred along the hollowed degraded channel and that endothelial cells populated the channel walls, forming hollow tubes that could predicate 3D cell microtubule formation.

It is worth noting that at the higher laser powers (e.g. >150 mW) and slower writing speeds (e.g. 0.25 mm/s), the bottom of the channel degraded completely, accompanied by high cell death (Supplementary Figure S4). Despite the increase in cell death, the outputted structure retained a curved valley floor that cells within the hydrogel (and possibly not in the initial area of cell death) migrated and adhered to, forming a 3D cell layer on the peaks and troughs.

## Discussion

Photolabile materials include those that respond to a specific wavelength of light (e.g. UV) to trigger a chemical or physical process such as degradation or crosslinking. Spatial degradation of a UV-responsive material was previously explored for directing 3D cellular responses, however, one disadvantage of this system is the relatively long timescale–in *minutes*–to achieve patterning^14^. Moreover, utility of shorter wavelengths (<400 nm) can be damaging to cellular content due to higher light absorption by water. Here we demonstrated hydrogel patterning with NIR light as a more benign way to pattern hydrogels as NIR can penetrate water or tissues up to several centimeters, making it an ideal candidate for tissue engineering applications^19^. Gold nanorods efficiently absorb NIR light and release this energy in the form of phonons (i.e. heat), called the *photothermal effect*, thus providing a mechanism for thermally denaturing the surrounding collagen matrix. Laser exposure parameters control the extent of this denaturation, which presumably alters mechanical properties of the hydrogel. It is widely known that matrix stiffness plays a crucial role in determining cell fate, such as cell migration and stem cell differentiation^20^.

Importantly, PEGylated gold nanorods at our concentration (5.45 nM) were not cytotoxic to cells for the duration of the experiments, which is in line with previous literature^21^. They may interact with cell membranes and can even be endocytosed, however, which may play a role in altering RNA, thus expression levels should be noted when considering a certain cell type or intended application^22^,^23^. Though we did not explore this option, the nanorod-cell interaction provides an interesting mechanism for local drug delivery. Nanorods can be functionalized with proteins and drugs and can be triggered to release their payload by NIR light (via the photothermal effect) or by becoming endocytosed^24^.

We previously explored the photothermal heating platform for patterning stiffness changes on a hydrogel substrate in 2D, however, to our knowledge, this concept has not yet been explored in 3D hydrogel patterning^16^. Additionally, it was previously demonstrated that hydrated collagen (similar to our collagen hydrogel) denatures at 55 °C^25^. Thus, it can be interpreted that our patterns are momentarily (i.e. for *milliseconds*) heating up to this temperature during patterning before returning to sink conditions of 37 °C. Below a certain threshold (e.g. patterning at 50 mW and 2.0 mm/s) we see neither a visible pattern nor any cell response (data not shown). Therefore we believe the collagen is not denaturing in this instance.

As with any new fabrication/materials/ process, finding an application for NR induced hydrogel degradation is key. We found that the right combination collagen material (e.g. 4 mg/ml), laser writing speed, and laser power allowed for fabrication of 3D channels inside a pre-made collagen encapsulating cell network with low cell death (<10%). Using an automated XY stage attached to the laser microscope (which controls the movements of the laser beam) enabled the patterning of parallel channels f or which cells migrated, aligned, and in the base of endothelial cells, formed hollow tubes similar to vascular tube formation. Previously, only a few groups have reported guidance of tube formation using labor-intensive techniques such as micromolding and casting^26^,^27^.

One such model that stands to benefit from this novel process is an angiogenesis model, whereby the creation of 3D endothelial tubes and subsequent seeding of a second cell type (e.g. cancer) can provide a facile means of studying angiogenesis between the two cell populations. Another possibility is to implement this platform with more complex vascular structures in hopes of guiding a biomimetic cellular organization (e.g. bifurcation) *in vitro*. It would also be advantageous to assess the perfusibility of these hollow tubes following patterning, cell migration, and tube formation. Lastly, this patterning technique could be employed for 3D cell patterning of other tissue types that demonstrate 3D alignment *in vivo* – including cardiac, muscle, and neural.

## Conclusion

The described platform allows for the patterning of internal channels in collagen hydrogels *in situ*, in this case enabling endothelial recruitment, alignment, and tube formation. Alignment of nearby cells can be visualized as early as 1 day post-patterning, while migration and tube formation may take up to 14 days. This platform has broad applications in *in vitro* cell patterning and can be applied to a host of light-responsive materials in addition to collagen. Furthermore, more complex patterns, and additionally other cell types, could be implemented to achieve a broader degree of 3D cell patterning for tissue engineering applications.

## Methods

### Materials

For gold nanorods, hydrochloroauric acid (HAuCl_4_), silver nitrate (AgNO_3_), sodium borohydride (NaBH_4_), and L-ascorbic acid were purchased from Sigma-Aldrich and cetyltrimethylammonium bromide (CTAB) was purchased from CalBioChem (EMD Millipore). Milli-Q water (18.2 Ω, MilliPore) was used in all synthesis steps. mPEG-thiol (5 kDa) (Nanocs) was used in nanorod surface modification. For hydrogels, collagen I, High Concentration (8.7 mg/mL) (VWR) was purchased.

### Gold Nanorod Synthesis and Surface Modification

Gold nanroods were synthesized and using a seed-mediated growth and surface-modification methodology as previously described with some modifications^28^. 7.5 mL 0.1 M of CTAB was mixed with 250 μL 0.01 M HAuCl_4_, followed by addition of 600 μL 0.01 NaBH_4_. Seeds formed after 2 minutes of mixing. The growth solution was prepared by mixing 40 mL of 0.1 M CTAB, 1.7 mL 0.01 M HAuCl_4_, 250 μL of 0.01 AgNO_3_, and 270 μL of 0.1 M L-ascorbic acid, followed by 420 μL of the seed solution. Nanorods formed after several hours. Nanorods were surface modified by first centrifuging twice at 15,000 *g*, removing the supernatant and resuspending in diH_2_O, followed by adding mPEG-SH dropwise and allow to gently mix for 2 hours. Nanorods were again pelleted and washed in diH_2_O to remove excess reactants and sterilized through a 0.22 μm filter for later use. Final nanorod concentration was determined to be 5.45 e-9 M by absorbance readings at its plasmon resonance (~800 nm).

### Cell Culture

Bend3 mouse endothelial cells were used for cell culture experiments. Bend3 were grown in EGM-2 media (Lonza) and passaged several times after thawing. In preparation for collagen gel experiments, cells were trypsinized in 0.25% trypsin-EDTA, pelleted and resuspended in EGM-2 at various concentrations (1.0 million/mL up to 5 million/mL).

### Gelation of Collagen-nanorod Hydrogels

Collagen gels were formed using the manufacturer’s instructions. Briefly, an ice-cold mixture of 1.04 μL of 1N NaOH, 10 μL 10× dPBS, and 10 μL of nanorods at their final concentration was prepared. Next, 33 μL of either diH_2_O or cells at various concentrations (in EGM-2) was added, again kept at 4 degrees. 46 μL of stock Collagen I (8.7 mg/mL) was added to the solution and pipetted slowly to mix the contents without producing air bubbles. The mixed solution was then added to 35 mm glass-bottom dishes with 10 mm wells (#0 cover glass, *In Vitro* Scientific) and placed in the incubator (37 degrees C, 5.0% CO_2_) for 30 minutes. Following gelation, 2 mL of warmed EGM-2 media was added for cell culture.

### *In Vitro* Hydrogel Patterning with bend3 Endothelial Cells

Gels were immediately used for patterning following gelation and addition of warmed media. The glass dishes were placed on an automatic stage atop an inverted microscope (Olympus). A femtosecond laser beam (100 femtoseconds, 800 nm wavelength, 80 MHz, Coherent) was used to pattern the samples. The laser was focused through the laser objective lens (10×, NA 0.45) and onto the gel sample. Patterns were drawn inside the hydrogels by varying the focal plane of the beam (in z-direction) and according to digital masks designed on the computer, with controlled writing speeds (mm/s) using the stage controller (MS2000, ASI). Average power of the laser beam was modulated using an attenuator, varying the power from 100–290 mW, read by a power meter (Coherent Fieldmax).

## Additional Information

**How to cite this article**: Hribar, K. C. *et al*. Three-dimensional direct cell patterning in collagen hydrogels with near-infrared femtosecond laser. *Sci. Rep*. **5**, 17203; doi: 10.1038/srep17203 (2015).

## Supplementary Material

Supplementary Information

## Figures and Tables

**Figure 1:**
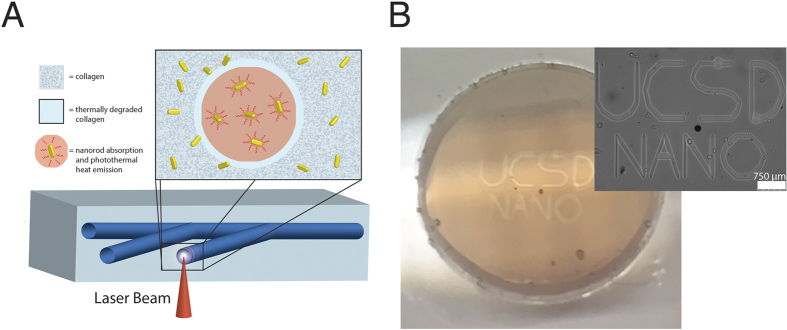
Schematic of the patterning process and resulting cell response. (**A**) A near-infrared (NIR) laser is focused inside the optically clear hydrogel and triggers the photothermal degradation of the collagen internally according to a computer-generated design, thereby creating channels. (**B**) Image of gel within the glass dish, with outset showing the corresponding brightfield image.
