# Supplementary material for: Three-dimensional direct cell patterning in collagen hydrogels with near-infrared femtosecond laser
Source: Sci Rep. 2015 Nov 25;5:17203. doi: 10.1038/srep17203 (PMC4658636; doi:10.1038/srep17203)
Supplement: Supplementary Information [file srep17203-s1.doc]

**Supplementary Information**

Three-dimensional direct cell patterning in collagen hydrogels with near-infrared femtosecond laser

Kolin C. Hribar ^1^, Kyle Meggs ^1^, Justin Liu ^2^, Wei Zhu ^1^, Xin Qu ^1^, Shaochen Chen ^1^*

^1^Department of NanoEngineering, University of California San Diego, La Jolla, CA, 92093-0448.

^2^ Materials Science and Engineering Program, University of California San Diego, La Jolla, CA

* chen168@eng.ucsd.edu


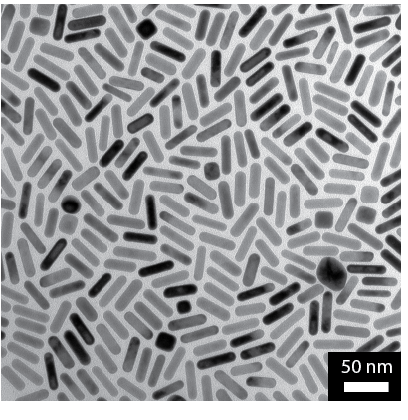


**Supplementary Figure 1**

Transmission electron microscopy (TEM) image of PEG-gold nanorods, showing >90% nanorods in relation to total nanoparticles (which also may include nanospheres and nanocubes).


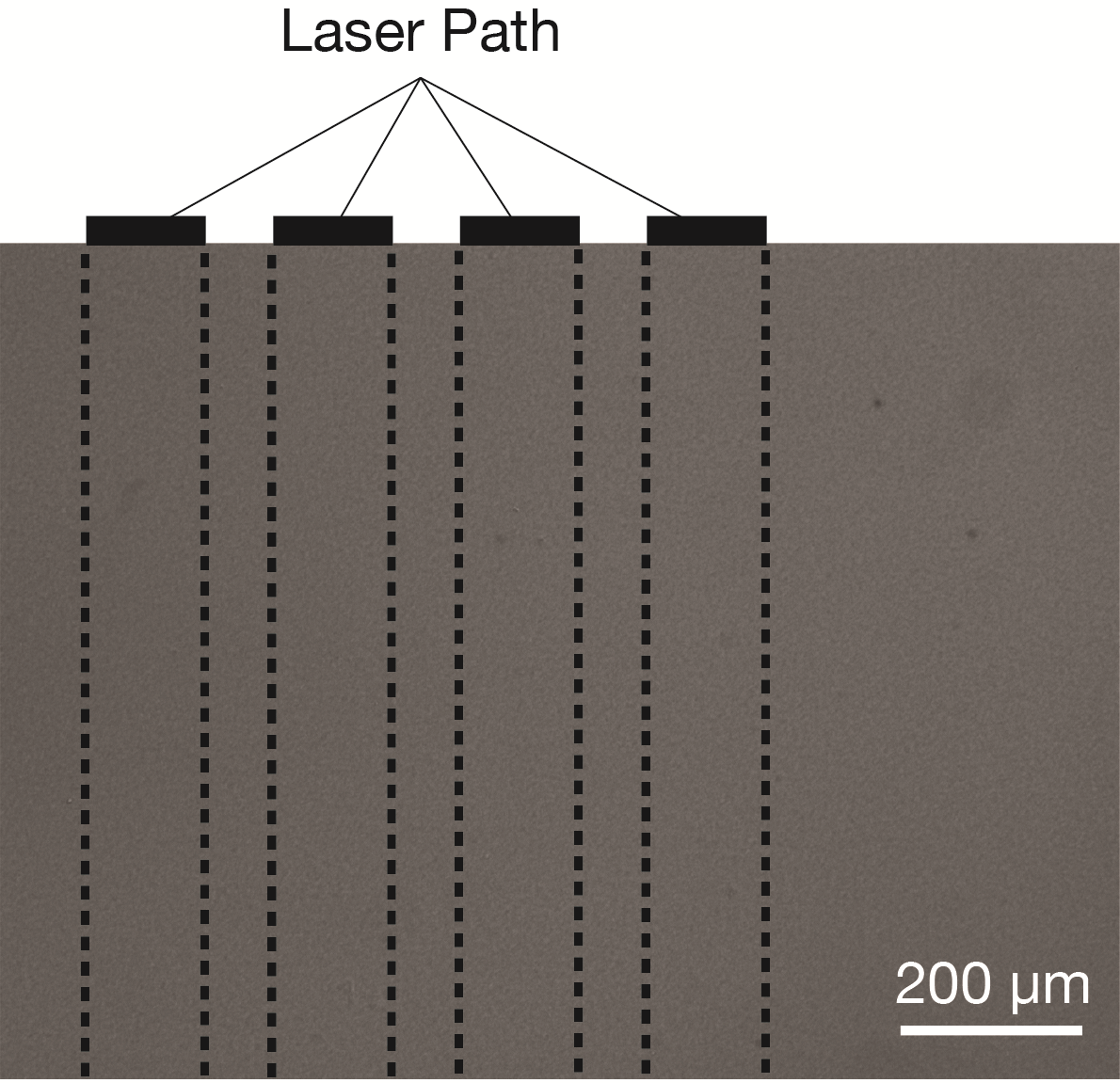


**Supplementary Figure 2**

Top view of a collagen hydrogel without nanorods showing no response to NIR light at maximum power and slow writing speeds (290 mW, 0.25 mm/s, respectively).


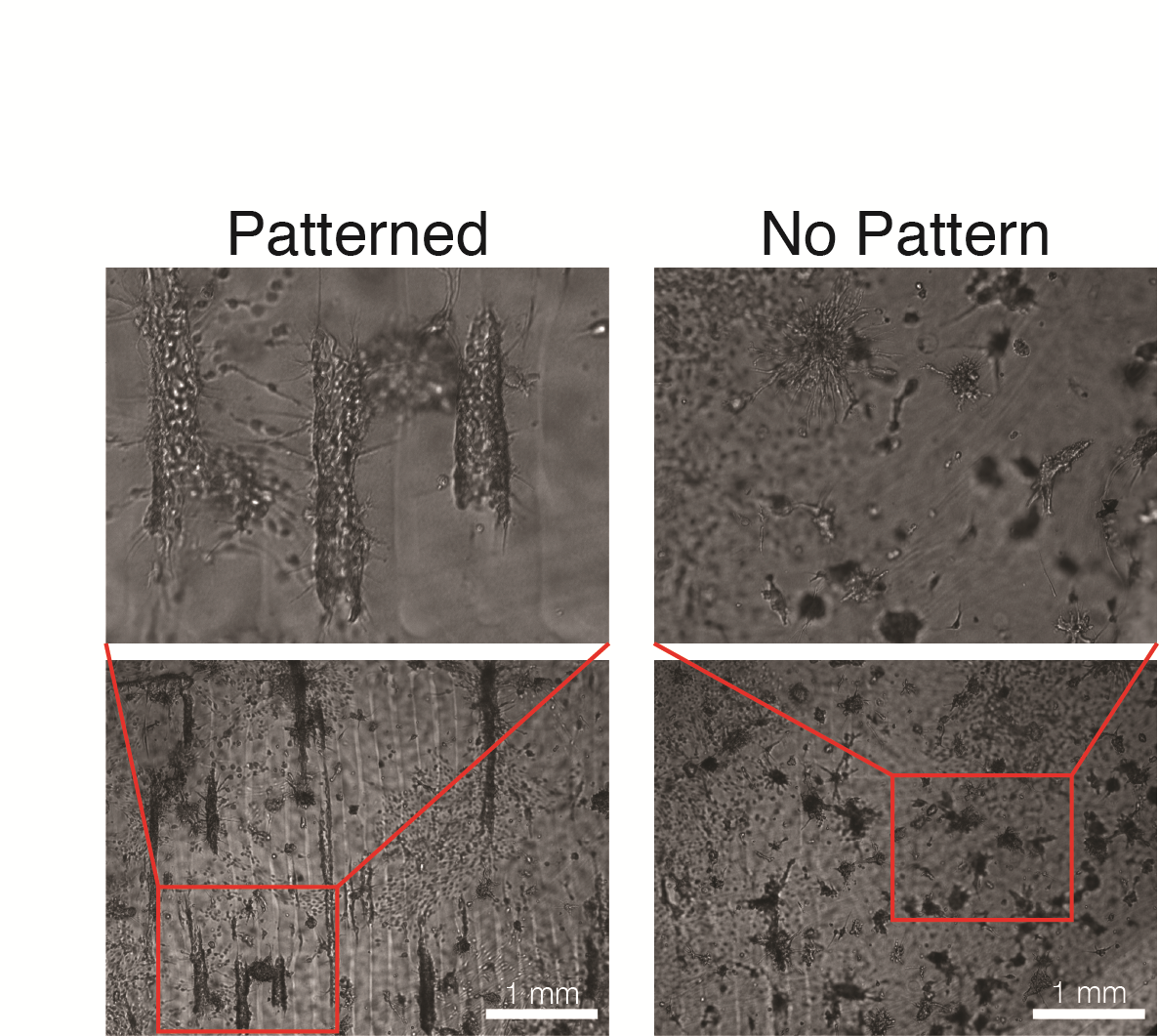


**Supplementary Figure 3**

Cell response to patterned and unpatterned collagen gels with gold nanorods after 14 days of culture.


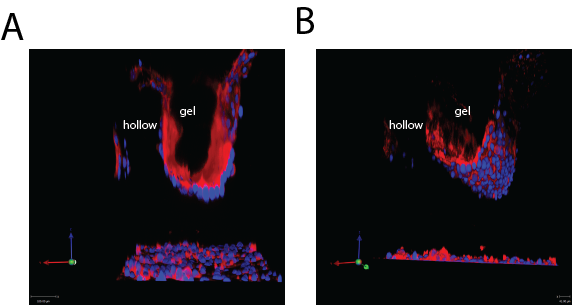


**Supplementary Figure 4**

Using a higher power and slower writing speed allows for complete denaturation of the collagen hydrogel on the bottom part of the hydrogel where the gel first comes into contact with the NIR laser. This figure depicts the side (A) and underneath (B) view of the gel upon 14 days of incubation with endothelial cells after exposure to 100mW laser intensity and 0.25 mm/s writing speed. The hollow areas show where the laser passed through. The cells migrate and adhere to the peaks and troughs of the patterned collagen.
